# A PD-L2-based immune marker signature helps to predict survival in resected pancreatic ductal adenocarcinoma

**DOI:** 10.1186/s40425-019-0703-0

**Published:** 2019-08-29

**Authors:** Yiyin Zhang, Jin Xu, Jie Hua, Jiang Liu, Chen Liang, Qingcai Meng, Miaoyan Wei, Bo Zhang, Xianjun Yu, Si Shi

**Affiliations:** 1 Department of Pancreatic Surgery, Fudan University Shanghai Cancer Center, Shanghai, China; 2 Department of Oncology Shanghai Medical College, Fudan University, Shanghai, China; 3 Pancreatic Cancer Institute, Fudan University, Shanghai, China; 4 Shanghai Pancreatic Cancer Institute, Shanghai, China

**Keywords:** Pancreatic ductal adenocarcinoma, PD-L2, Immune marker, Prognosis, TGF-β2

## Abstract

**Background:**

Programmed cell death protein 1 (PD-1) is a key immune checkpoint that regulates peripheral tolerance and protects against autoimmunity. Programmed death ligand-2 (PD-L2) is a less studied ligand to PD-1 and has yet to be fully explored, especially in pancreatic ductal adenocarcinoma (PDAC).

**Methods:**

In this study, we performed immunohistochemistry to detect the PD-L2, CD3, CD8, transforming growth factor-β2 (TGF-β2) and FOXP3 levels in paraffin sections from 305 patients with resected PDAC as a training set. Expression levels of intratumoral and stromal immune markers were compared in relation to survival using Kaplan-Meier curves, random survival forest model and survival tree analysis. A multivariable Cox proportional-hazards model of associated markers was used to calculate the risk scores.

**Results:**

PD-L2 was expressed in 71.5% of PDAC samples and showed strong correlations with CD3+, CD8+ T cells and FOXP3+ regulatory T cell densities. High levels of intratumoral PD-L2 and FOXP3 were related to poor survival; only stromal FOXP3 overexpression was associated with worse prognosis. Four patterns generated from survival tree analysis demonstrated that PD-L2^low^_stromal_FOXP3^low^ patients had the longest survival, while PD-L2^high^_intratumoral_CD3^low^ patients had the shortest survival (*P* <  0.001). The area under the curve was 0.631(95% confidence interval (CI): 0.447–0.826) for the immune marker-based signature and 0.549 (95% CI: 0.323–0.829; *P* <  0.001) for the clinical parameter-based signature, which was consistent with the results in the validation set including 150 patients (*P* <  0.001). A higher risk score indicated shorter survival and could serve as an independent prognostic factor. PD-L2 was also showed associated with TGF-β2 and other immune molecules based on bioinformatics analysis.

**Conclusions:**

Our work highlighted PD-L2 as a promising immunotherapeutic target with prognostic value combined with complex tumor infiltrating cells in PDAC.

**Electronic supplementary material:**

The online version of this article (10.1186/s40425-019-0703-0) contains supplementary material, which is available to authorized users.

## Introduction

Pancreatic ductal adenocarcinoma (PDAC) has a poor prognosis, with a 5-year survival rate of approximately 8% [1]. Although surgical resection remains the only curative treatment, most patients still receive systemic chemotherapy as for the prevention of recurrence. Compared to conventional chemotherapy, cancer immunotherapies have achieved remarkable success in a wide range of solid tumors [2, 3].

Programmed cell death protein 1 (PD-1) is an immune checkpoint that regulates peripheral tolerance and protects against autoimmunity. PD-1 has two ligands, namely, programmed death ligand-1 (PD-L1) and programmed death ligand-2 (PD-L2). PD-L1 is upregulated on tumor-infiltrating lymphocytes (TILs) and some solid tumors, while PD-L2 is limited to macrophages, dendritic cells (DCs) and hematologic malignancies. Immune checkpoint blockade of PD-1 has shown promising initial efficacy in advanced PDAC, with a 70% disease control rate in 11 included patients [4]. However, only patients with PDAC who are microsatellite instability-high and have mismatch repair deficiency are suitable for PD-1 blockade treatment in current clinical practice; this population accounts for approximately 2% of all PDAC populations [5]. Therefore, it is important to identify specific patient groups that would benefit from immunotherapies and to find other approaches involving therapeutic combinations.

PDAC is known for a desmoplastic stroma which mainly contains cancer-associated fibroblasts, inflammatory cells and fibronectin. Myofibroblast depletion alters immune gene expression, which increases the sensitivity of PDAC to checkpoint blockade, but simultaneously results in enhanced tumor aggressiveness, as the action of the stroma relies on the context of PDAC. The results showed that the stroma and TILs affect PDAC cells in a complex way, indicating that therapeutics for PDAC should not neglect the underlying connection between the surrounding stromal composition and lymphocytes. High PD-L2 expression was found associated with increased PD-1+ TILs, indicating its functional role in the tumor microenvironment [6]. TILs have value in assessing prognosis and evaluating the outcomes of immunotherapies, and the dysfunction in TILs may result in early metastasis and worse survival. The cell densities of certain subtypes of T cells, such as CD3+ T cells, CD8+ T cells and FOXP3+ regulatory T cells (Tregs), determine their immunoactive and immunosuppressive effects on the tumor microenvironment.

To date, few studies have examined the correlations of different types of immune cell infiltrates with clinical parameters, and the prognostic and therapeutic significance of PD-L2. Thus, we analyzed the expression of 4 essential immune markers in PDAC, including the immune checkpoint molecule PD-L2, mature T cell marker CD3+, cytotoxic antitumor T cell marker CD8+ and immunosuppressive Treg marker FOXP3+, to clarify their interaction and prognostic potential.

## Materials and methods

### Clinical information of patients with PDAC

A total of 455 patients with primary PDAC who underwent surgical resection at the Fudan University Shanghai Cancer Center (FUSCC) were included in this study (training set: January 2011–July 2015, *n* = 305; validation set: August 2015–May 2016, *n* = 150). None of the patients included in our study received any anti-cancer treatment, including chemotherapy and radiotherapy, before surgical resection. Tumor grade and stage were defined according to the 8th edition of the American Joint Committee on Cancer (AJCC) staging system. Patients from training set were followed up for survival status until December 2016, and patients from the validation set were until Nov 2018, and their medical records were reviewed. This study was approved by the Institutional Research Ethics Committee.

### Immunohistochemistry and evaluation

Formalin-fixed and paraffin-embedded 4 μm thick serial tumor sections were deparaffinized in xylene and rehydrated in ethanol. Next, 3% H_2_O_2_ was used to block endogenous peroxidase for 15 min. High-pressure heat-induced antigen retrieval was conducted in pH 6.0 citric acid (Wuhan Servicebio Technology, China) for 5, 8, 8, 10, 20 and 20 min for PD-L2, CD3, CD8, transforming growth factor-β2 (TGF-β2), PD-L1 and FOXP3 respectively. After 1 h blocking with 5% normal goat serum, mouse monoclonal anti-PD-L2 (MAB1224–100, 1:1000, R&D, USA), mouse monoclonal anti-CD3 (60181–1-Ig, 1:800, Proteintech, USA), rabbit monoclonal anti-CD8 (ab93278) 1:500 and mouse monoclonal anti-TGF-β2 (ab36495) 1:100 (both Abcam, USA), rabbit monoclonal anti-PD-L1 (13684S) 1:100 and rabbit monoclonal anti-FOXP3 (98377S) 1:200 (both Cell Signaling Technology, USA) antibodies were incubated with tissue slides overnight at 4 °C. After 3 washes in phosphate-buffered saline, the sections were incubated with secondary antibodies (GTVisionTM III Detection System/Mo&Rb, GK500710, Gene Tech, China) for 1 h at room temperature and washed for 3 times. Following 3,3-diaminobenzidine coloration (GK500710, Gene Tech, China) at a dilution of 1:200 (GK500710, Gene Tech, China) and with hematoxylin counterstaining, the sections were dehydrated in ethanol and xylene. All samples were successfully analyzed for the expression of PD-L1, PD-L2, TGF-β2, CD3, CD8 and FOXP3 without any loss of tumor tissue. The staining intensity of PD-L1 and PD-L2 in PDAC cells were scored as 0 (negative), 1 (weak), 2 (moderate) and 3 (strong), and the number of positive cells was also recorded intratumorally. The evaluation of stromal PD-L2 expression was classified as negative/positive. TGF-β2 was evaluated using the immunoreactive score proposed by Remmele and Stegner [7]. Intratumoral and stromal CD3, CD8 and FOXP3 expression was quantified in 20x fields using Cellsens standard software (Olympus, Japan). The mean counts of 3 fields were used for statistical analysis. The optimal cut-off point was set using X-tile (Yale University, USA). All scoring was performed by 2 experienced pathologists.

### Gene set enrichment analysis

Gene Set Enrichment Analysis (GSEA) version 3.0 (Broad Institute, USA) [8] was used to analyze for patient samples from The Cancer Genome Atlas (TCGA) based on high or low expression of PD-L2 to investigate potential mechanism in molecular signatures. We chose 1000 times of permutations and Affymetrix as the chip platform to calculate the *P* value and false discovery rate q-value. All basic and advanced fields were set to default values.

### Random survival forest and risk score models

We constructed a random survival forest (RSF) model using variables selected by variable importance (VIMP) and the minimal depth. The VIMP threshold was used to estimate the predictive values of the included variables and sort the variables into the RSF model according to their importance. Minimal depth was inversely correlated with the predictive value of variables. Survival tree analysis was performed based on the variables selected by VIMP and minimal depth. The branches were drawn using the log-rank splitting rule, which selected the optimal variables related to survival and the terminal nodes were estimated using Kaplan-Meier analyses [9]. A risk score model was produced by integrating the expression level of immune markers selected by the RSF model and their corresponding coefficients derived from multivariate analyses, as follows: risk score = (0.637 * intratumoral PD-L2) - 0.437 * intratumoral CD3 + (0.499 * stromal FOXP3). Reference signatures such as T stage, N stage, AJCC stage and differentiation were divided into high/low levels and scored as 0/1, and these scores were multiplied by the associated coefficients to generate a reference score model as follows: reference score model = (0.911 * AJCC stage) + (0.510 * grade differentiation) + (0.633 * T stage) + (1.087 * N stage) [10, 11]. The areas under time-dependent receiver operating characteristic (ROC) curves (AUCs), ranging from 0.5 to 1.0, were used to evaluate the quality of the scores [12].

### Statistical analysis

Correlations between intratumoral and stromal expression levels of immune markers were determined by paired *t* tests. Linear regression and *χ*^*2*^ tests were performed to evaluate the correlations, and the log-rank test was employed to compare the survival curves based on immune marker expression. Comparisons between groups were performed using the *χ*^*2*^ test. The Cox proportional hazards model was employed for multivariate analysis by including all statistically significant covariates (*P* <  0.1) from the univariate Cox model (backward Wald). All analyses were carried out using the ‘randomForestSRC’ and ‘survivalROC’ packages in by R studio (version 3.5.0, R development core team), SPSS version 22 (SPSS Inc., IBM) and GraphPad (version 5.01, GraphPad Software, Inc.). *P* <  0.05 was considered statistically significant.

## Results

### Expression levels of PD-L2, CD3, CD8, and FOXP3 in the training set

**Fig. 1:**
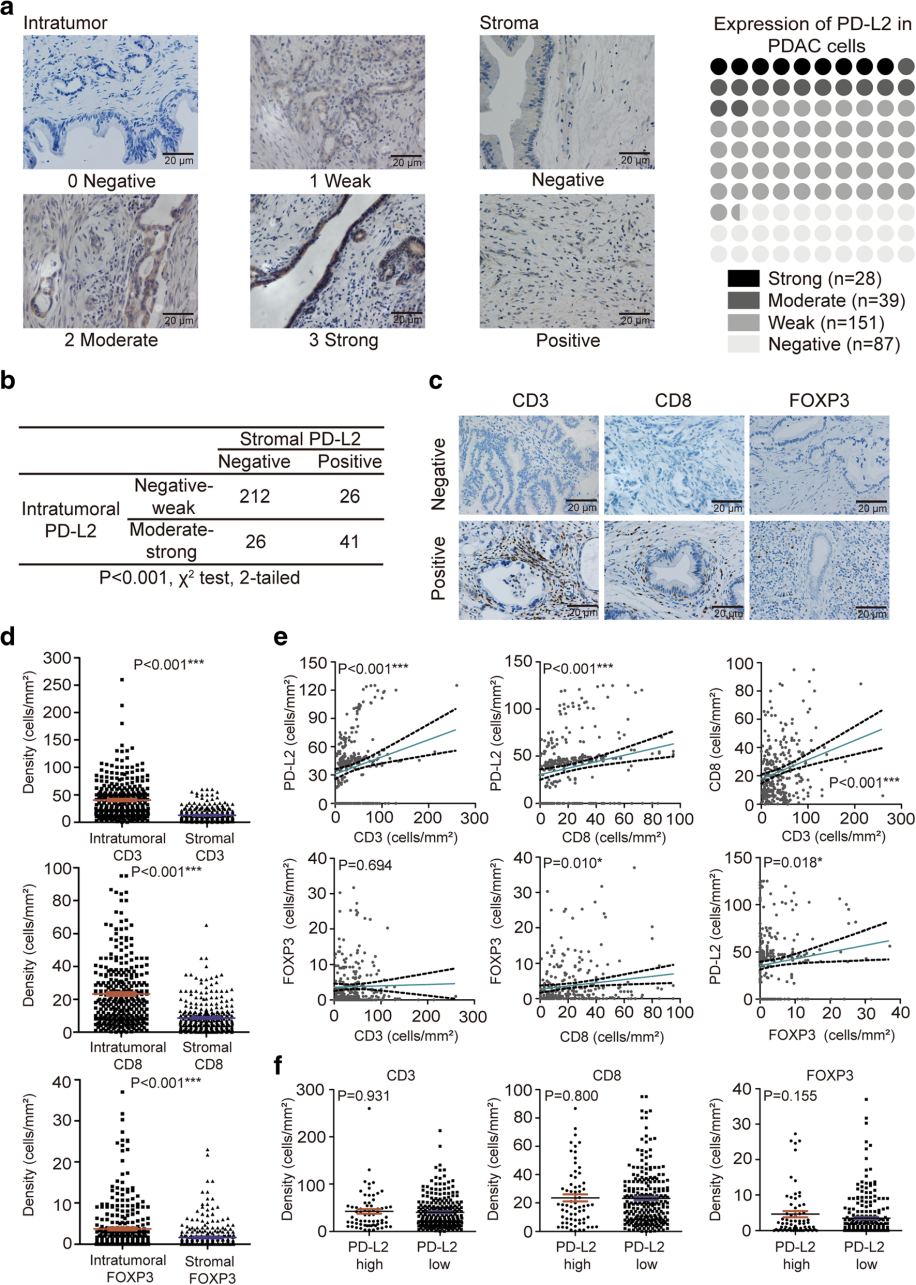
PD-L2 in PDAC. **a** Stratification of PD-L2 expression in PDAC cells (scales bar: 20 μm) and in the stroma. **b** ﻿Statistical results of correlation between intratumoral and stromal PD-L2 using the *χ*^*2*^ test. **c** Expression of CD3, CD8 and FOXP3 in PDAC TILs. **d** Comparison between intratumoral and stromal densities of CD3+, CD8+ and FOXP3+ T cells in PDAC using paired *t* tests. **e** Scatter plots with linear regression for 4 immune markers using Pearson’s correlation. **f** Relation among densities of CD3+, CD8+, and FOXP3+ T cells based on PD-L2 expression levels using the Mann-Whitney *U* test. * *P* < 0.05; **P < 0.01; ***P < 0.001

Membranous or cytoplasmic PD-L2 expression was observed in 218 (71.5%; Fig. 1a) patients, and the stromal PD-L2 expression was positive in 67 patients (Fig. 1b). The cut-off values for intratumoral counts of CD3+, CD8+, FOXP3+ T cells were 51.0, 41.0 and 8.3, respectively, while those for stromal CD3+, CD8+, and FOXP3+ T cell counts were 6.0, 20.3 and 0.3, respectively (Fig. 1 c).

Strong correlations were discovered among the densities of PD-L2 tumor cells, CD3+ T cells, CD8+ T cells, and FOXP3+ Tregs in the tumor and stroma (all *P* <  0.001; Fig. 1d). The densities of CD3+ T cells and CD8+ T cells were significantly higher than the densities of FOXP3+ Tregs in both the intratumoral and stromal areas. The densities of intratumoral CD3+ T cells, CD8+ T cells and FOXP3+ Tregs were positively correlated with PD-L2 expression based on linear regression analyses (*P* <  0.001, *P* <  0.001, and *P* = 0.018, respectively; Fig. 1e). Moreover, positive correlations were observed between densities of intratumoral CD8+ and CD3+ T cells (*P* <  0.001), and FOXP3+ Tregs (*P* = 0.010). However, there was no significant correlation was observed between intratumoral CD3+ T cells and FOXP3+ Tregs (*P* = 0.694; Fig. 1e).

### Associations of PD-L2, CD3, CD8, and FOXP3 expression levels with clinicopathological features in PDAC

In total, 305 patients, aged 34 to 81 years (median, 63 years), were analyzed in the training set. The median follow-up time was 24.8 months. By the end of this study, 243 (79.8%) deaths were recorded. The detailed patient characteristics were shown in Table 1 and Additional file 1 Table S1. The results of univariate and multivariate analysis by the Cox proportional hazards model are shown in Table 2. Multivariate analysis indicated that T3 stage, N2 stage, AJCC stage III and low differentiation were associated with poorer prognosis (*P* ≤ 0.001). Intratumoral PD-L2 [hazard ratio (HR) 1.892, 95% confidence interval (CI): 1.402–2.552; *P* <  0.001], CD3 (HR 0.646, 95% CI: 0.482–0.865; *P* = 0.003), and FOXP3 (HR 1.704, 95% CI: 1.215–2.389; *P* = 0.002) and stromal CD3 (HR 1.319, 95% CI: 1.012–1.721; *P* = 0.041) were independent prognostic factors.

**Table 1:** Intratumoral PD-L2 expression and TILs in relation to clinicopathologic characteristics of PDAC

Intratumoral expression													
		PD-L2			CD3			CD8			FOXP3		
	N	Low (0–1)	High (2–3)	*P*	Low	High	*P*	Low	High	*P*	Low	High	*P*
Sex													
Male	158	125	33	0.540	113	45	0.805	129	29	0.066	131	27	0.502
Female	147	112	35		107	40		131	16		126	21	
Age													
< 60	106	86	20	0.340	76	30	0.138	86	20	0.139	88	18	0.663
≥60	199	152	47		144	55		174	25		169	30	
Location													
Head	181	136	45	0.348	130	51	0.913	153	28	0.885	147	34	0.030
Body	62	49	13		44	18		53	9		59	3	
Tail	62	52	10		46	16		54	8		51	11	
Grade													
Well	16	14	2	0.361	10	6	0.677	14	2	0.908	13	3	0.941
Moderate	175	140	35		127	48		150	25		148	27	
Low	114	85	29		83	31		96	18		96	18	
Tumor stage													
T1	51	43	8	0.494	33	18	0.252	40	11	0.212	45	6	0.694
T2	176	135	41		133	43		149	27		147	29	
T3	78	60	18		54	24		70	8		65	13	
Node stage													
N0	154	125	29	0.020	109	45	0.343	137	17	0.007	133	21	0.562
N1	108	79	29		76	32		83	25		88	20	
N2	43	40	3		35	8		35	8		36	7	
AJCC stage													
I	124	103	21	0.154	90	34	0.274	109	15	0.071	108	16	0.509
II	138	101	37		95	43		111	27		113	25	
III	43	34	9		35	8		40	3		36	7	

*TILs* tumor infiltrating lymphocytes, *PDAC* pancreatic ductal adenocarcinoma

**Table 2:** Univariate and multivariate analysis of overall survival factors.

	N	Overall survival			
		Univariable analysis		Multivariable analysis	
		HR (95% CI)	*P*	HR (95% CI)	*P*
All	305				
Age (years)			0.588		
<60	106	Ref			
≥60	199	0.929 (0.711-1.213)			
Sex			0.916		
Male	158	Ref			
Female	147	1.007 (0.888-1.142)			
T stage			< 0.001		< 0.001
T1-2	227	Ref		Ref	
T3	78	1.744 (1.333-2.361)		2.065 (1.542-2.767)	
N stage			< 0.001		< 0.001
N0-1	262	Ref		Ref	
N2	43	2.487 (1.754-3.526)		3.049 (2.127-4.373)	
AJCC stage			< 0.001		< 0.001
I-II	262	Ref		Ref	
III	43	2.487 (1.754-3.526)		3.049 (2.127-4.373)	
Grade			0.001		< 0.001
Well and moderate differentiation	191	Ref		Ref	
Low differentiation	114	1.536 (1.187-1.989)		1.632 (1.255-2.124)	
Intratumoral PD-L2			< 0.001		< 0.001
Low expression	238	Ref		Ref	
High expression	67	1.858 (1.387-2.487)		1.892 (1.402-2.552)	
Intratumoral CD3			0.07		0.003
Low cell densities	220	Ref		Ref	
High cell densities	85	0.768 (0.577-1.022)		0.646 (0.482-0.865)	
Stromal CD3			0.053		0.041
Low cell densities	119	Ref		Ref	
High cell densities	186	1.295 (0.997-1.683)		1.319 (1.012-1.721)	
Intratumoral CD8			0.143		
Low cell densities	260	Ref			
High cell densities	45	1.292 (0.917-1.821)			
Stromal CD8			0.066		0.372
Low cell densities	272	Ref		Ref	
High cell densities	33	0.663 (0.427-1.028)		0.814 (0.517-1.279)	
Intratumoral FOXP3			0.006		0.002
Low cell densities	257	Ref		Ref	
High cell densities	48	1.580 (1.138-2.193)		1.704 (1.215-2.389)	
Stromal FOXP3			0.008		0.132
Low cell densities	292	Ref		Ref	
High cell densities	13	2.157 (1.227-3.790)		1.647 (0.860-3.153)	
Risk score			< 0.001		< 0.001
Low score	228	Ref		Ref	
High score	77	2.047 (1.549-2.706)		1.836 (1.379-2.444)	

### Construction of prognostic model for predicting overall survival in PDAC

**Fig. 2:**
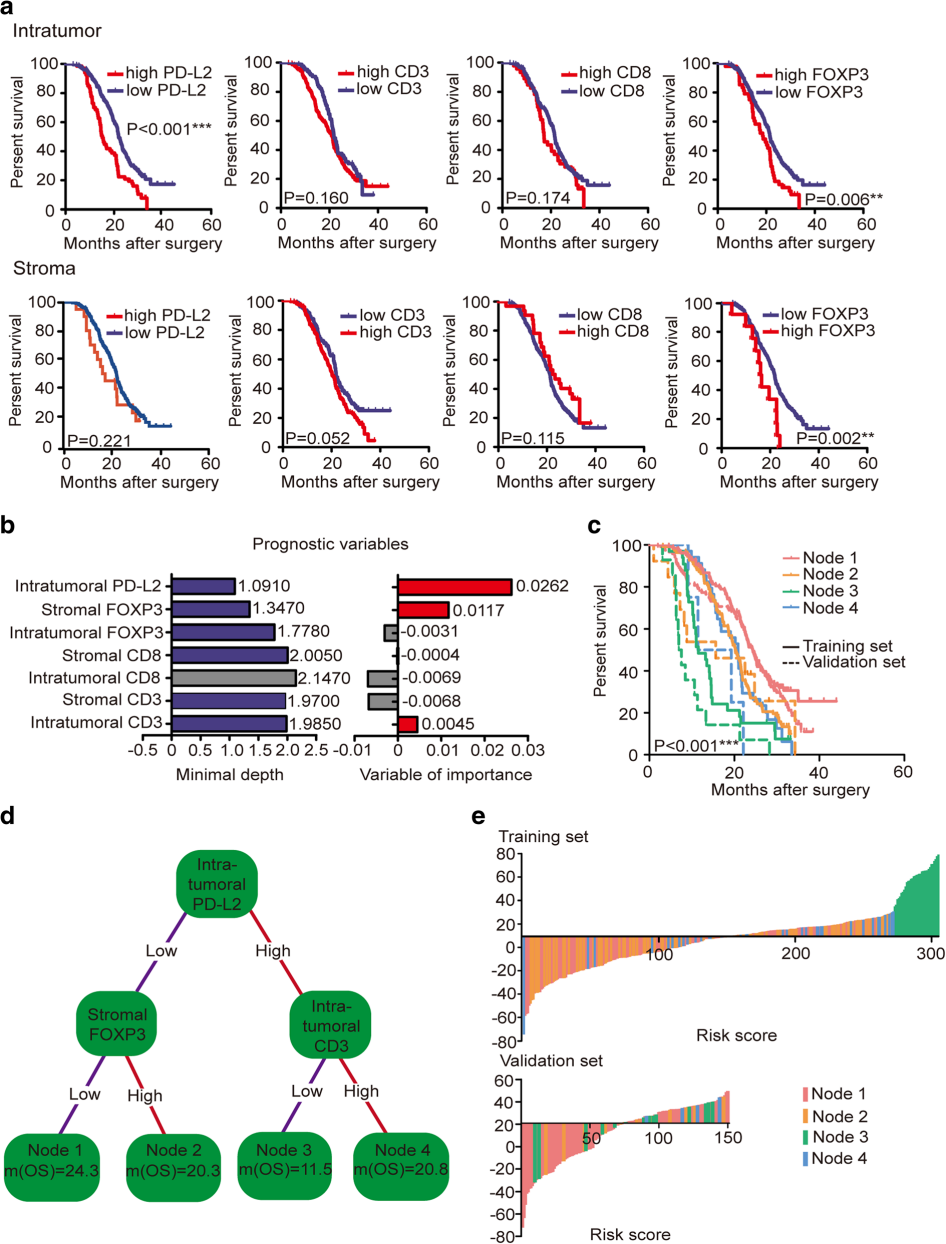
Prognostic association between different immune markers and OS. **a** Log-rank test showing associations between OS and immune markers in the tumor and stroma. **b** The RSF model using the minimal depth and VIMP of prognostic variables in predicting OS. The variables most related to survival had smaller minimal depth and greater importance. The minimal depth ruled out the maximum variable, and VIMP ruled out variables with negative properties (colored in gray). **c** Survival curves of 4 nodes in the training set and the validation set. **d** A survival tree was generated using variables selected by the RSF model. Each variable has 2 nodes per branch depending on survival. **e** Waterfall plot showing relevant risk scores of four immune marker-based signatures in the training set and the validation set

A high prevalence of single immune markers, such as FOXP3, can lead to PDAC progression and poor prognosis, but given that the immune system plays both anti- and pro-tumorigenic roles, immune modulations of the tumor microenvironment involving immunoactive and immunosuppressive molecules should not be neglected [13]. We found that high intratumoral PD-L2 expression was associated with worse overall survival (OS) than low PD-L2 expression (17.8 months vs 24.3 months; HR 1.858, 95% CI: 1.387–2.487; *P* <  0.001; Fig. 2a). The simple classification of CD3, CD8 and FOXP3 expression into 2 groups based on PD-L2 expression was not directly associated with prognosis (*P* = 0.931, *P* = 0.800, and *P* = 0.155, respectively; Fig. 1 f); thus, we next sought to determine the potential relationships among PD-L2, CD3, CD8 and FOXP3. We hypothesized that immune molecules influence prognosis differently with different extents of tumor and stromal expressions. To verify this hypothesis, we included the expression of PD-L2 and intratumoral and stromal expression of CD3, CD8 and FOXP3 in a RSF model to select the survival-related variables. In the minimal depth analysis, intratumoral CD8 had the maximum value with a minimal depth of 2.1470; thus, it was excluded from the RSF model (gray color). In the variable of importance analysis, PD-L2 was identified as the most influential variable (VIMP = 0.0262), while intratumoral CD8 and FOXP3 and stromal CD3 and CD8 had values of − 0.0069, − 0.0031, − 0.0068 and − 0.0069, respectively, and were all excluded from the RSF model due to their negative properties (gray). Thus, only intratumoral PD-L2 and CD3 (_intratumoral_CD3) and stromal FOXP3 (_stromal_FOXP3) were suitable for the construction of the RSF model and to complete the prognostic evaluation (Fig. 2b).

A regression tree showed that PD-L2^low^_stromal_FOXP3^low^ patients (Node 1) had better median survival than PD-L2^high^_intratumoral_CD3^low^ patients (Node 3; 24.3 months vs 11.5 months; *P* <  0.001; Fig. 2 d) and patients in the 2 other Nodes (PD-L2^low^_intratumoral_CD3^high^: 20.8 months; PD-L2^low^_stromal_FOXP3^high^: 20.3 months). These results initially confirmed our hypothesis that multiple immune markers interact between the tumor and stroma, explaining why studies of single immune markers had controversial results.

**Fig. 3:**
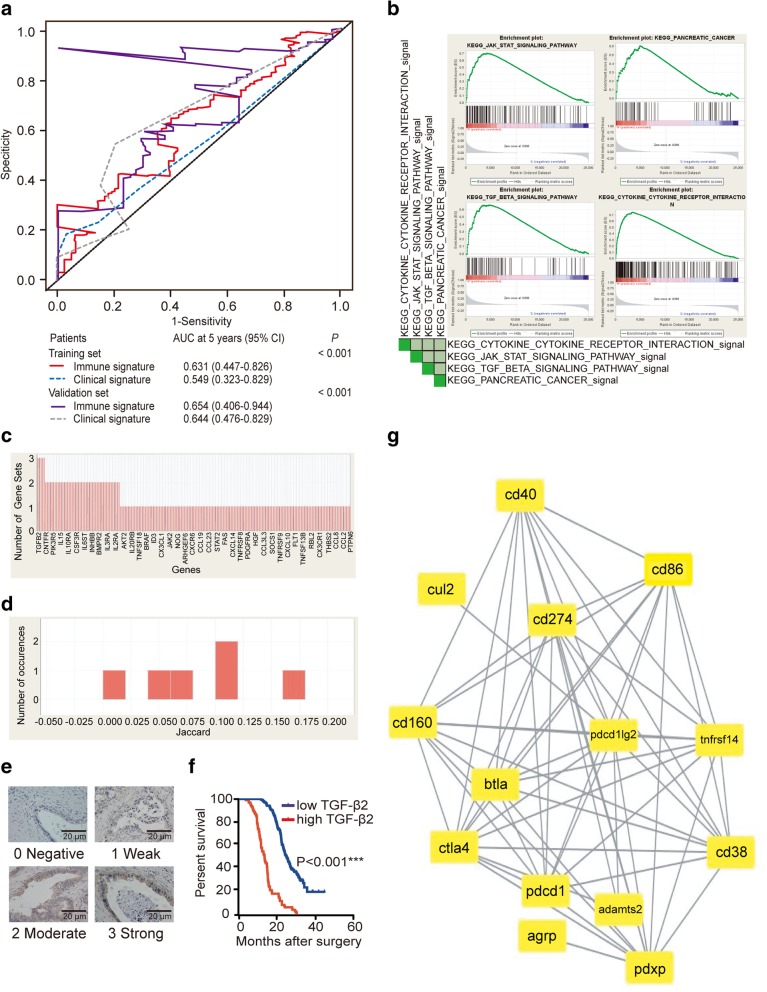
Validation of signatures for predicting survival and potential therapeutic use of PD-L2. **a** Time-dependent ROC curves and AUCs for 2 signatures predicting survival in the training set and the validation set. The red solid line and blue dashed line represent the immune marker-based model and the clinical parameter-based model in the training set, with AUCs of 0.631 (95% CI: 0.447–0.826) and 0.549 (95% CI: 0.323–0.829; *P* < 0.001), respectively. The purple solid line and gray dashed line represent the immune marker-based model and the clinical parameter-based model in the validation set, with AUCs of 0.654 (95% CI: 0.406–0.944) and 0.644 (95% CI: 0.476–0.829; *P* < 0.001), respectively. **b** Signatures in C2 were determined using PD-L2 expression by GSEA. **c** Four gene sets with enrichment scores greater than 0.60 and false discovery rates less than 0.25 were chosen for the leading edge analysis. TGF-β2 is the most overlapping gene among the leading edge genes. **d** The paired Jaccard index is above 0.02, indicating that most of the paired subsets have coincident parts. **e** Stratification of TGF-β2 expression in PDAC cells (scale bar: 20 μm). **f** Log-rank test results showing associations between OS and TGF-β2 in PDAC. **g** PD-L2 and its relationship with other immune molecules

We further built a risk score model based on variables selected from the RSF model. Clinical parameters such as T stage, N stage, AJCC stage, grade, intratumoral PD-L2, CD3, and FOXP3 and stromal CD3, CD8 and FOXP3 were all included in the multivariable analysis using the Cox proportional hazards model (*P* <  0.1; Fig. 2e). Covariates were extracted from the Cox proportional hazards model to construct immune marker-based prognostic and clinical parameter-based risk score models. A waterfall plot intuitively showed that patients in Node 3 with poorer prognoses had higher risk scores, mainly ranging from 34 to 79. Patients in Node 1 with better prognoses were observed mostly clustered on the left side of the plot, while patients in Nodes 2 and 4 were scattered on both sides of the plot. Furthermore, we performed multivariate analysis using a Cox proportional hazards model including risk scores, essential clinical features and immune variables with *P* <  0.05 from univariate analyses. The multivariate Cox regression showed that the risk score was an independent prognostic factor for resected PDAC patients in our study, and higher risk scores were associated with shorter survival (HR 1.836, 95% CI: 1.379–2.444; *P* <  0.001). Sensitivity and specificity comparisons were performed via time-dependent ROC curve analysis of immune marker-based and clinical parameter-based prognostic signatures. AUC values obtained from ROC analyses were compared between the 2 signatures and were 0.549 (95% CI: 0.323–0.829) for the clinical parameter-based signature and 0.631 (95% CI: 0.447–0.826) for the immune marker-based signature (*P* <  0.001; Fig. 3 a). Therefore, the immune marker-based signature is a more powerful prognostic index than the clinical parameter-based signature.

### Validation of the immune marker-based prognostic signature in PDAC

In an effort to validate the immune marker-based prognostic signature, we further performed immunohistochemistry for CD3, CD8, FOXP3 and PD-L2 in 150 patients as an independent cohort. The clinical characteristics of patients in the validation cohort are shown in Additional file 1 Table S2. Four terminal nodes were generated: patients in Node 1 (PD-L2^low^_stromal_FOXP3^low^) had the longest survival of 25.1 months compared to 7.2 months for patients in Node 3 (PD-L2^high^_intratumoral_CD3^low^), showing good concordance with the training set. The risk score was calculated using the equation described in the methods, with Node 1 patients mostly scattered on the left of the plot (Fig. 2e). The results of univariate and multivariate analyses in the validation cohort are shown in Additional file 1 Table S3 and confirmed our data from the training set. The validation set revealed an AUC of 0.654 (95% CI: 0.406–0.944) for the immune marker-based signature and 0.644 (95% CI: 0.476–0.829) for the clinical parameter-based signature, which were statistically significant (*P* <  0.001; Fig. 3a). We also performed a log-rank test in the 4 nodes, and the validation set showed similar survival trends to the training set (Fig. 2c).

### PD-L2 and TGF-β2

To better understand the relationship between PD-L2 and other molecules involved in the tumor microenvironment, we analyzed PD-L2 expression levels based on the TCGA database in Cytoscape (National Institute of General Medical Sciences, USA) and C2-curated gene sets in GSEA. Of the 4726 gene sets in C2, the high PD-L2 expression-associated signatures “KEGG_CYTOKINE_CYTOKINE_RECEPTOR_INTERACTION”,” KEGG_JAK_STAT_SIGNALING_PATHWAY”,” KEGG_TGF_BETA_SIGNALING_PATHWAY” and” KEGG_PANCREATIC_CANCER” displayed considerable normalized enrichment scores with *P* <  0.008 (Fig. 3b). Leading edge analysis showed that 4 signatures had high overlaps, and most of the numbers of the occurrences had a Jaccard index > 0.02 (Fig. 3d). Fig. 3c presents a strong connection among the 4 signatures, and *TGF-β2* was recognized as the most overlapping gene, showing that it might play an important role in the high PD-L2 expression. For further validation of the possible interaction between TGF-β2 and PD-L2, we performed immunohistochemistry on samples from the original 305 patients in the training set. The rate of positive TGF-β2 expression in PDAC was 91.1%, with 64.4% weak, 27.7% moderate, and 7.9% strong expression (Fig. 3e). High expression of TGF-β2 predicted poorer survival than did low expression (12.9 months vs 24.3 months, *P* <  0.001; Fig. 3f) and was positively correlated with PD-L2 expression (*P* <  0.001; Additional file 1: Table S4). *TNFRSF14*, *CD86*, *CD38*, *BLTA*, *CTLA-4*, *CD160* and *CD160* were directly connected in the molecular network of PD-L2 in Cytoscape in Fig. 3g.

## Discussion

To overcome the therapeutic bottleneck in PD-1 and PD-L1-based immunotherapy and to improve the accuracy of immune markers in predicting the survival of patients with resected PDAC, we analyzed whether the combination of multiple immune indicators in both intratumoral and stromal components might predict postoperative survival in PDAC. Our findings highlighted the prognostic value of PD-L2 in PDAC, and the use of an immune marker-based signature provided better survival predictions than the use of a single immune marker. Moreover, although PD-L2 has not been as fully explored in immunological research as PD-L1, it is still strongly related to immunoregulation and tumor progression and provides valuable prospects for future treatment.

PD-L1, one of the important cosuppression molecules expressed on macrophages, DCs and many types of cancer cells, was detected with an approximately 49.4% positive expression rate in PDAC cells. The conclusions of whether the expression of PD-L1 in PDAC influences TNM stage, perineural invasion, lymphocytic infiltration and patient outcomes vary across studies [14–16]. Moreover, anti-PD-1/PD-L1 blockade monotherapy has shown poor efficacy in treating PDAC [17]. Therefore, in the beginning of this study design, we aimed to explore the possible reason for the failure of PD-L1 immunotherapy and the relationship between PD-L1 and the complex tumor microenvironment in PDAC. We performed immunohistochemistry on samples from 305 patients in the training set for intratumoral PD-L1 expression at first and failed to find a significant relation with survival (*P =* 0.202; Fig. S1a). The stromal expression of PD-L1 (9.5%; Additional file 1: Figure. S1b) was related to the intratumoral PD-L1 expression (*P* <  0.001) but was not related to survival outcomes (*P* = 0.445; Additional file 1: Fig. S1a). PD-L2 expression is induced by interferon gamma (IFN-γ) at the protein and mRNA levels in the T cell-inflammatory tumor microenvironment in cancer and can appear independently of PD-L1 [18, 19]. Emerging studies have examined correlations between immune cell infiltration and clinical parameters and the prognostic and therapeutic significance of PD-L2 in other cancer types [19–21]; thus, we performed preliminary experiments on tissue slides and surprisingly found a relation between PD-L2 and OS in PDAC.

The establishment of prognostic models to distinguish patients with better prognosis often depends on TNM staging, micro-RNA signatures and metabolic markers in PDAC [22–24]. However, just as the immunoscore in colon cancer provides a reliable estimate of the risk of recurrence, simple and effective immune system-based prognostic signatures that can be applied in clinical practice are urgently needed [25]. PDAC is commonly regarded as an immunologically “cold” tumor due to its lack of response to checkpoint blockade treatments, but as the TIL repertoire presents abundant overlaps between each other in different regions of the same pancreatic tumor, the discovery of TIL enrichment in our study suggested that the adaptive immune response in PDAC might involve immunoactive, cytotoxic and immunoregulatory T cell subgroups in the intratumorally and in inflammatory stromal region [26]. Our results showed that PD-L2 was overexpressed in 71.5% of patients, and approximately 20% of all patients had high PD-L2 expression and had a shorter median OS than patients with low PD-L2 expression. Most of the patients expressed CD3+, CD8+ and FOXP3+ T cells, but not all the densities of TILs were directly associated with prognosis. Although cancer-associated fibroblasts were previously reported to activate deregulating signals that reduced T cell infiltration, a novel computational imaging technology combined with multiple immune-labeling markers failed to identify correlations of T-cell accumulation with collagen-I and αSMA^+^ fibroblasts [27, 28]. These results implied that the relationships between the stroma and T cells might be more complicated than previously believed. A recent study suggested that a high frequency of PD-L1+ CD4+ CD25+ Tregs in the tumor microenvironment could increase the number of PD-1+ CD8 Tregs and induce a more lethal effect of TILs by PD/PD-L1 blockade therapy [29]. Stromal expression of PD-L2 was also evaluated during the exploration of a possible relationship between PD-L2 and TILs in PDAC. However, we later excluded stromal PD-L2 from further analysis due to its lower positive rate and fewer strong staining results than intratumoral PD-L2 (22.0% vs 71.5%; Fig. 1a) and its lack of association with patient survival outcomes (*P =* 0.221; Fig. 2a). We did not include the evaluation of tumor-associated macrophages (TAMs) and myeloid-derived suppressor cells (MDSCs) in our study because the positive rate of TAMs remained approximately 2–3%, although they are highly related to PD-L1 expression (*P* <  0.001) [30]. PD-L2 is expressed at relatively lower levels in tumor-infiltrating MDSCs than PD-L1 in several tumor types, and immune tolerance induction of PD-L2 on MDSCs has rarely been studied [31, 32]. Additionally, the detection of TAMs and MDSCs required CD68, CD163, HLA-DR, CD33, CD11b, CD14 and CD15, which would increase the difficulty of developing a simple and practical prognostic signature. Therefore, we hypothesized that intratumoral and stromal TILs combined with intratumoral PD-L2 expression might have value in prognostic prediction.

The regression tree intuitively showed that incorporating intratumoral CD3 and stromal FOXP3 could highlight the prognostic potential of PD-L2 in PDAC, which was more accurate than the clinical parameter-based signature, as validated using time-dependent ROC curves. The waterfall plot of risk scores showed that compared to patients in other subgroups, patients in the PD-L2^high^_intratumoral_CD3^low^ subgroup had the worst survival, while patients in the PD-L2^low^_stromal_FOXP3^low^ subgroup had the best outcome. These findings indicated that PD-L2 might participate in the modulation of intratumoral CD3+ and stromal FOXP3+ cells. Moreover, combined variables showed better prognostic predictions than single markers for minimizing the false-negative rate.

An immune phenotype is not directly linked to a certain immunotherapy response because the tumor-immune microenvironment is vital for promoting the efficacy of current immunotherapies [33]. In our study, the GSEA results suggested that the most significant changes in pathways and molecules in C2-curated gene sets based on PD-L2 expression were “TGF-BETA SIGNALING PATHWAY”, “JAK-STAT SIGNALING PATHWAY”, “CYTOKINE-RECEPTOR INTERACTION” and “PANCREATIC CANCER”, with TGF-β2 being the most differentially expressed molecule. High PD-L2 expression is strongly related to TGF-β2, which is induced by differentiation and growth arrest signals, but little research has been performed on TGF-β2 and cancer. Thus, we analyzed TGF-β2 expression in the training set and identified high expression of TGF-β2 as an unfavorable prognostic factor (12.9 months vs 24.3 months, *P* < 0.001) with a positive correlation with PD-L2 expression (*P* < 0.001). As TGF-β2 is known to be capable of inhibiting the activation of T cells, B cells, and inducing Tregs, we further explored its correlation with intratumoral and stromal CD3, CD8 and FOXP3. We found that TGF-β2 was positively correlated with intratumoral CD3 (*P* = 0.004; Table S4), indicating that the poor prognosis of patients with high PD-L2 expression may be related to immunoregulation by TGF-β2 in tumor immunity. Inhibition of TGF-β2 is also observed in the local inflammatory environment, as TGF-β2 antisense gene-modified therapeutic vaccine, known as belagenpumatucel-L, showed improved survival within 12 weeks of platinum-based chemotherapy in non-small cell lung cancer patients who received prior radiation [34]. OT-101, a TGF-β2 inhibitor, was shown to result in a major survival benefit in patients with advanced pancreatic cancer [35]. TGF-β2 suppression led to the elevation of interleukin (IL)-8, IL-15 and ﻿human hepatocyte growth factor, which also ranked on the top of the list of intersections of gene sets in our analysis, and these findings suggest that immune checkpoint blockade in combination with TGF-β2 inhibitors might benefit patients with immune exhaustion signatures because high expression of TGF-β2 in Node 3 patients (PD-L2^high^_intratumoral_CD3^low^) is associated with poor prognosis (11.0 months vs 23.0 months, *P* < 0.001).

Notably, stromal cell types in the tumor microenvironment are more stable than tumor cells; thus, the use of immunohistochemistry to stain tumoral and stromal immune markers is a feasible method to establish a prognostic model for daily clinical practice. It is also feasible to use PD-L2 and other immune molecules to evaluate the efficacy of treatment. The B7–28 family consists of CD80, CD86, B7–1, B7–2, CD275, CD274 (PD-L1), PD-L2, B7-H4, BHNL2 and TNFRSF14. A Cytoscape network diagram in our study showed that PD-L2 in PDAC is closely related to the expression of PD-L1, CD86, TNFRSF14, PD-1, CD160 and CTLA-4, which are important for the regulation of immunodeficiency and autoimmune diseases [36]. The CD86 + 1057G/A polymorphism and AG (+ 1057, + 2379) haplotype are genetic risk factors for PDAC [37]. High tumoral expression of TNFRSF14 was associated with improved survival in PDAC, and binding of TNFRSF14 to BTLA or CD160 led to the inhibition of T cells [38]. Carcinoma-associated pancreatic fibroblasts promoted the expression of CTLA-4 and PD-1 in proliferating T cells, which contribute to immune evasion by inducing the expression of immune checkpoint inhibitors on CD4+ and CD8+ T cells in PDAC [39]. This study provides insights into the link between PD-L2 and other B7–28 family molecules to serve as indicators for the prognosis of immunotherapy. PD-L2 could also serve as a biomarker for treatment efficacy and have therapeutic value. Low levels of soluble PD-L2 and IL-2 and high levels of soluble IFN-γ were associated with grade 3/4 toxicities in non-small cell lung cancer treated with nivolumab. Circulating PD-L2 levels could help to identify patients with a high risk for severe toxicity from the beginning of immunotherapy, which is helpful for clinical practice, as it can alert physicians to closely observe these patients [40]. The activation of the JAK-STAT pathway promotes the expression of PD-L2, and the blockade of this activation can reverse the reduced production of IFN-γ. The IFN-γ pathway is also associated with PD-L2 enrichment in colorectal cancer, which indicates that the combination of IFN-γ pathway inhibitors and PD-L2 blockade might benefit PDAC patients. Ahmad SM et al. found that PD-L2-specific T cells reacted to autologous target tumor cells based on PD-L2 expression. The PD-L2-related vaccine could serve as a complementary therapy and immune checkpoint inhibitor because competitive therapy could work along both lines by preventing the inhibition of PD-L2-specific T cells at the tumor site [41].

Despite substantial computational evidence for the prognostic potential of immune marker-based signatures in PDAC, there are still some limitations in our study: 1) the immune molecules included in our study are not the only molecules related to survival; thus, other molecules might also have an impact on survival and 2) although bioinformatics analysis of PD-L2 suggested its potential in immunotherapy, further experimental validation is still needed to elucidate its function in PDAC.

## Conclusions

In summary, we identified immune marker-based prognostic signatures and risk scores consisting of PD-L2, intratumoral CD3 and stromal FOXP3 for survival prediction, and these signatures and risk scores were significantly associated with the OS of patients with PDAC. The immune marker-based prognostic signature was superior to the clinical parameter-based signature at survival prediction, and the risk score was an independent prognostic indicator. PD-L2 and TGF-β2 were positively correlated with each other and associated with poor prognosis, indicating that a combined inhibition of these factors might improve the immunotherapeutic efficacy. This study revealed that PD-L2 has potential future applications in immunotherapy and predictive value in PDAC.

## Additional file

Additional file 1: **Table S1.** Stromal PD-L2 expression and cell densities of TILs in relation to clinicopathologic characteristics of PDAC. **Table S2.** Clinicopathologic characteristics of PD-L2 expression and cell densities of TILs of PDAC in validation set. **Table S3.** Univariate and multivariate analysis of overall survival factors in validation set. **Table S4.** The correlation of TGF-β2 expression and immune markers in PDAC using 2-tailed *χ*2 test. **Fig. S1.** PD-L1 expression in PDAC. (PDF 877 kb)

## Abbreviations

AJCC: American Joint Committee on Cancer
APC: Advanced pancreatic cancer
AUC: Area under the curve
CI: Confidence interval
CIK: Cytokine-induced killer
DC: Dendritic cell
FUSCC: Fudan University Shanghai Cancer Center
GSEA: Gene set enrichment analysis
HR: Hazard ratio
IFN-γ: Interferon gamma
IL: Interleukin
MDSCs: Myeloid-derived suppressor cells
OS: Overall survival
PD-1: Programmed cell death protein 1
PDAC: Pancreatic ductal adenocarcinoma
PD-L1: Programmed death ligand-1
PD-L2: Programmed death ligand-2
ROC: Receiver-operating characteristic curve
RSF: Random survival forest
TAMs: Tumor-associated macropahges
TCGA: The Cancer Genome Atlas
TGF-β2: Transforming growth factor-beta 2
TILs: Tumor-infiltrating lymphocytes
Tregs: Regulatory T cells
VIMP: Variable importance
