# Supplementary material for: A PD-L2-based immune marker signature helps to predict survival in resected pancreatic ductal adenocarcinoma
Source: J Immunother Cancer. 2019 Aug 29;7:233. doi: 10.1186/s40425-019-0703-0 (PMC6716876; doi:10.1186/s40425-019-0703-0)
Supplement: Supplementary file 1 — Table S1. Stromal PD-L2 expression and cell densities of TILs in relation to clinicopathologic characteristics of PDAC. Table S2. Clinicopathologic characteristics of PD-L2 expression and cell densities of TILs of PDAC in validation set. Table S3. Univariate and multivariate analysis of overall survival factors in validation set. Table S4. The correlation of TGF-β2 expression and immune markers in PDAC using 2-tailed χ2 test. Fig. S1. PD-L1 expression in PDAC. (PDF 877 kb) [file 40425_2019_703_MOESM1_ESM.pdf]

Table S1. Stromal PD-L2 expression and cell densities of TILs in relation to clinicopathologic characteristics of PDAC.

		Stromal expression											
		PD-L2			CD3			CD8			FOXP3		
	N	Negative	Positive	*P*	Low	High	*P*	Low	High	*P*	Low	High	*P*
**Sex**													
Male	158	49	109	0.318	62	96	0.934	137	21	0.150	152	6	0.677
Female	147	38	109		57	90		135	12		140	7	
**Age**													
<60	106	30	76	0.950	39	67	0.561	92	14	0.327	103	3	0.366
≥60	199	57	142		80	119		180	19		189	10	
**Location**													
Head	181	50	131	0.897	83	98	0.952	162	19	0.500	176	5	0.060
Body	62	18	44		27	35		57	5		60	2	
Tail	62	19	43		28	34		53	9		56	6	
**Grade**													
Well	16	3	13	0.656	7	9	0.679	15	1	0.684	16	0	0.685
Moderate	175	50	125		71	104		154	21		167	8	
Low	114	34	80		41	73		103	11		109	5	
**Tumor stage**													
T1	51	21	30	0.072	21	30	0.922	44	7	0.071	51	0	0.203
T2	176	48	128		67	109		153	23		168	8	
T3	78	18	60		30	48		75	3		73	5	
**Node stage**													
N0	154	43	111	0.819	68	86	0.153	133	21	0.270	149	5	0.655
N1	108	30	78		35	73		99	9		102	6	

N2	43	14	29		16	27		40	3		41	2	
**AJCC stage**													
I	124	35	89	0.813	53	71	0.540	106	18	0.217	122	2	0.144
II	138	38	100		50	88		126	12		129	9	
III	43	14	29		16	27		40	3		41	2	

TILs tumor infiltrating lymphocytes, PDAC pancreatic ductal adenocarcinoma.

Table S2. Clinicopathologic characteristics of PD-L2 expression and cell densities of TILs of PDAC in validation set.

	Intratumoral expression													
		PD-L2			CD3			CD8			FOXP3			
	N	Low (0-1)	High (2-3)	*P*	Low	High	*P*	Low	High	*P*	Low	High	*P*
**Sex**													
Male	74	67	7	0.345	42	32	0.334	68	6	0.317	68	6	0.040
Female	76	65	11		49	27		66	10		61	15	
**Age**													
<60	53	44	9	0.165	36	17	0.179	46	7	0.456	44	9	0.437
≥60	97	88	9		55	42		88	9		85	12	
**Grade**													
Well	15	15	0	0.874	10	5	0.919	15	0	0.218	13	2	0.704
Moderate	98	83	15		58	40		87	11		85	13	
Low	37	34	3		23	14		32	5		31	6	
**Tumor stage**													
T1	38	37	1	0.065	19	19	0.113	37	1	0.108	36	2	0.008
T2	69	59	10		43	26		60	9		61	8	
T3	43	36	7		29	14		37	6		32	11	
**Node stage**													
N0	61	59	2	0.035	33	28	0.202	54	7	0.506	55	6	0.117
N1	68	55	13		44	24		60	8		58	10	
N2	21	18	3		14	7		20	1		16	5	
**AJCC stage**													
I	49	48	1	0.040	24	25	0.076	45	4	0.996	45	4	0.078
II	80	66	14		53	27		69	11		68	12	

III	21	18	3	14	7	20	1	16	5

TILs tumor infiltrating lymphocytes, PDAC pancreatic ductal adenocarcinoma.

Table S3. Univariate and multivariate analysis of overall survival factors in validation set.

	N	Overall survival			
		Univariable analysis		Multivariable analysis	
		HR (95% CI)	*P*	HR (95% CI)	*P*
**All**	150				
**Age (years)**			0.599		
<60	54	Ref			
≥60	96	0.906 (0.629-1.307)			
**Sex**			0.378		
Male	74	Ref			
Female	76	1.173 (0.822-1.674)			
**T stage**			< 0.001		0.008
T1-2	107	Ref		Ref	
T3	43	2.281 (1.557-3.341)		1.724 (1.152-2.578)	
**N stage**			< 0.001		< 0.001
N0-1	128	Ref		Ref	
N2	22	2.417 (1.491-3.920)		3.484 (2.051-5.920)	
**AJCC stage**			< 0.001		< 0.001
I-II	128	Ref		Ref	
III	22	2.417 (1.491-3.920)		3.130 (1.859-5.269)	
**Grade**			< 0.001		< 0.001
Well and moderate differentiation	113	Ref		Ref	

Low differentiation	37	2.128 (1.425-3.177)		2.616 (1.712-3.996)	
**Intratumoral PD-L2**			< 0.001		< 0.001
Low expression	132	Ref		Ref	
High expression	18	4.635 (2.731-7.867)		4.591 (2.608-8.082)	
**Intratumoral CD3**			< 0.001		0.004
Low cell densities	91	Ref		Ref	
High cell densities	59	0.490 (0.335-0.717)		0.559 (0.375-0.833)	
**Stromal CD3**			0.142		
Low cell densities	74	Ref			
High cell densities	76	0.767 (0.538-1.093)			
**Intratumoral CD8**			0.057		0.012
Low cell densities	134	Ref		Ref	
High cell densities	16	1.695 (0.985-2.917)		2.198 (1.198-4.062)	
**Stromal CD8**			0.139		
Low cell densities	82	Ref			
High cell densities	68	0.763 (0.533-1.092)			
**Intratumoral FOXP3**			< 0.001		< 0.001
Low cell densities	129	Ref		Ref	
High cell densities	21	3.113 (1.909-5.078)		2.816 (1.681-4.716)	
**Stromal FOXP3**			< 0.001		0.06
Low cell densities	133	Ref		Ref	
High cell densities	17	2.562 (1.528-4.296)		1.707 (0.978-2.979)	
**Risk score**			< 0.001		0.001

Low score	82	Ref	Ref
High score	68	3.665 (2.331-5.761)	2.695 (1.519-4.781)

HR hazard ratios, Ref reference.

Covariates with $P < 0.1$ in univariable analysis were included in multivariable analysis. A $P < 0.05$ in multivariable analysis was considered significant.

Table S4. The correlation of TGF-β2 expression and immune markers in PDAC using 2-tailed χ^2 test.

		TGF-β2		
		Negative-weak	Moderate-strong	*P*
**Intratumoral PD-L2**	Negative-weak	177	61	< 0.001
	Moderate-strong	29	38	
**Intratumoral CD3**	Low	138	82	0.004
	High	68	17	
**Stromal CD3**	Low	84	35	0.363
	High	122	64	
**Intratumoral CD8**	Low	181	79	0.063
	High	25	20	
**Stromal CD8**	Low	180	92	0.144
	High	26	7	
**Intratumoral FOXP3**	Low	179	78	0.069
	High	27	21	
**Stromal FOXP3**	Low	200	92	0.092
	High	6	7	

Fig. S1 PD-L1 expression in PDAC.

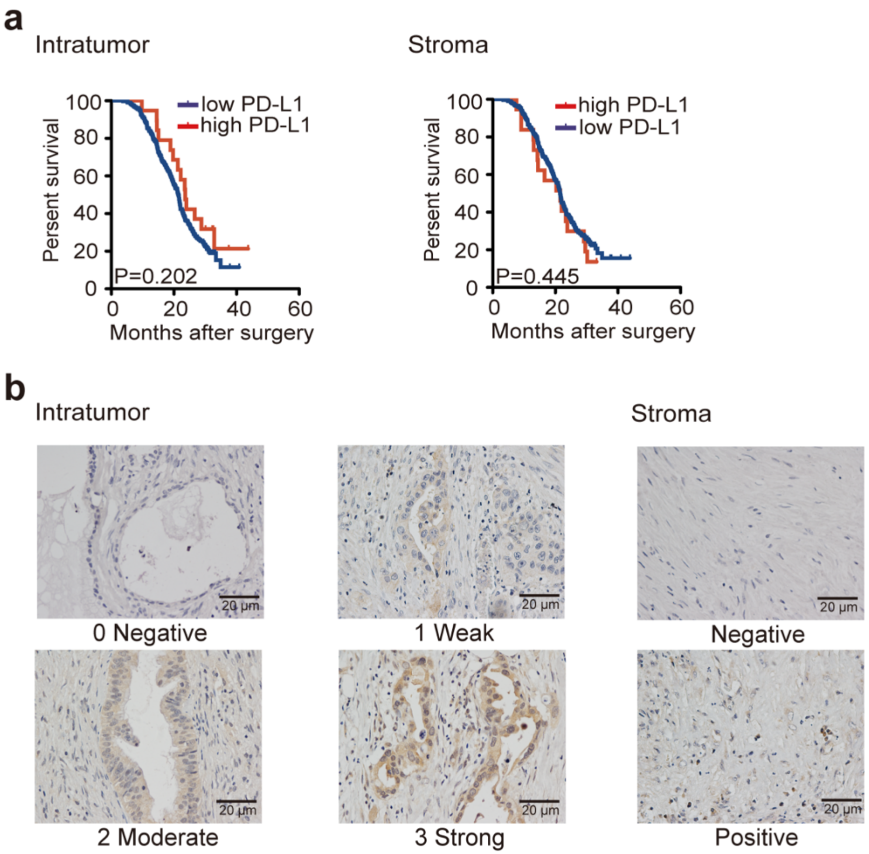

a Log-rank test showing associations between OS and PD-L1 expression in the tumor and stroma. **b** Stratification of PD-L1 expression in PDAC cells (scales bar: 20 μm) and in the stroma.
